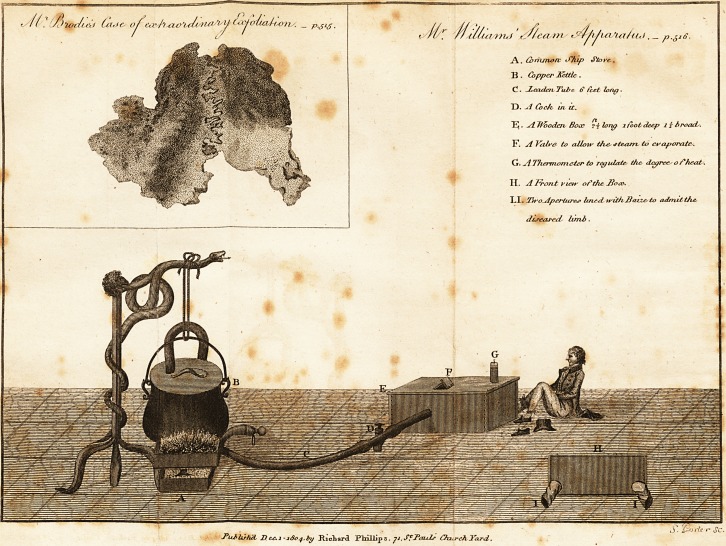# Mr. Williams's Steam Apparatus

**Published:** 1804-12-01

**Authors:** R. Williams

**Affiliations:** Surgeon. H. M. S. Northumberland, off Ferrol


### *To the Editors of the Medical and Physical Journal.*

GENTLEMEN,

ENCLOSED I beg leave to send you a drawing of a Steam Apparatus, which I suggested might be of material service in the cure of malignant ulcer, in the inflammatory stage; and from the evident success which attended it here during a tedious continuance of the disease of twelve months and upwards, I would strongly recommend the trial of it to all my brother officers in the Navy, who may have such an obstinate complaint to contend with. It can always be used at sea; the only necessary article to be procured on shore is, a leaden tube about six feet long, with a cock in it, as described in the *plate*, by which and the valve on the top of the box, the degree of heat required can be most correctly regulated with the assistance of a small hand thermometer. I found its good effects on many occasions when inflammation was seated on any of the extremities, in removing irritation, abating pain, and relaxing the surrounding integuments. The time necessary to keep the diseased part in the box, I found, was about one hour morning and evening, and the degree of heat about 100; if continued longer, it was liable to produce languor and syncope. Immediately on being removed from the steam, warm emollient poultices were applied applied with a view to assit in promoting suppuration, and which I found, from repeated proofs, to answer fully my expectations, by causing the sloughs to separate much quicker than could be expected, and by that means preventing many denudations and tedious exfoliations of the tibia. I am, &c.

R. WILLIAMS, Surgeon.

*H. M. S. Northumberland, off Ferrol, October* 30, 1804.

## Figures and Tables

**Figure f1:**